# Video Games and Other Online Activities May Improve Health in Ageing

**DOI:** 10.3389/fmed.2018.00008

**Published:** 2018-01-29

**Authors:** Marios Kyriazis, Elisavet Kiourti

**Affiliations:** ^1^ELPIS Foundation for Indefinite Lifespans, Larnaca, Cyprus; ^2^University of Cyprus, Nicosia, Cyprus

**Keywords:** hormesis, stress response, video games, information exposure, literacy practices

## Introduction

Humans are highly adaptable organisms. Our environment is now increasingly more digital, and we need to assimilate a vast amount of information, much of which requires us to act (1). This has an impact on our neuronal stress response mechanisms, which are activated in order to minimize any disturbance to our homeostasis, or more precisely to our homoeodynamic space (2). Therefore, function is dynamically maintained within healthy limits.

In this paper, we propose that by exposing ourselves to informative, co-interactive digital platforms which are defined by a variety of “Information-that-requires-action in time and on demand,” we are subjected to beneficial positive stress which upregulates neuronal function. This neuronal upregulation has a positive impact on the rest of the somatic repair processes which may reduce age-related degeneration (3). Beneficial positive stress is invoked when the information is important or significant, in the sense that it entices the organism to act, and thus adapt (i.e., reach a state which is functionally better compared to the previous one). When such information reaches the neuron, it acts as a cognitive stimulus which induces the neuron to upregulate its metabolism in order to process this new information (4). It has been argued that such exposure to novel digital information can influence our somatic repair processes through hormetic events (3). Hormesis is a widespread phenomenon in nature that is defined by a non-linear “low dose stimulation, high dose inhibition” principle. This means that exposure to a relatively weak stimulus may positively challenge the organism and upregulate the stress response. This results in health benefits, whereas an excessive, suboptimal, or prolonged exposure may result in damage and disease (5). Hormetic challenges (positive stress) may be used in order to reduce age-related dysfunction in general (6).

## Discussion

### Video Games in Later Life

There is increasingly strong evidence that online video games have beneficial cognitive effects in older adults (7). For instance, video game training improves several aspects of cognitive functioning (8), such as reaction time, memory, and attention span, as well as general cognitive control and multitasking (9). Complex 3-D video games improve hippocampal-associated memory (10) and also physical parameters, such as postural balance and muscle strength (11), particularly those games that concentrate on strategy (12). Video games may also be useful in dementia (13). One way these activities may effect health enhancement is through a hormetic mechanism. In this case, the instigation of hormesis is based on a continual state of cognitive novelty, when one operates at the “outer edge of their comfort zone,” performing mental activities that are “challenging but do-able.” In practice, this translates as an activity that provokes rather than annoys, and it is pleasantly frustrating.

### Cognitive Stimulation through Video Games

In the case of online video games, we find an example which is at the intersection where digital information, human brain, and human biology interact. Many thousands of players all over the world participate in virtual online worlds and interact with a variety of video game genres. Globally, gamers play competitively and cooperatively in their communities of practice (14, 15), in a way that is beyond just entertainment. Video games are complex digi-social microcosms which are constructed by social conventions and rules that may be different from actual world rules. For example, the rules may defy the physical law of gravitation and others, such as mortality and social conventions. These microcosms are carriers of complex layers of meaning, reflecting a certain set of ideologies about society and its power relationships. These virtual microcosms induce hormetic health benefits (16). It is not just a matter of being physically able to perform an action, but it is also an opportunity to interact with new kinds of text genres that require different literacy skills than the conventional ones encountered when we passively read a book. These new text genres are very relevant not only for the individual player but also for the rest of the team because the players can use the information to initiate actions which influence the behavior of other players. Players are becoming agents who provide meaning of the text within the game (17, 18). The linguistic interactions and overall performance of the player is also relevant, because these engage the player into debates with other players and thus strengthen social bonds. These social bonds may, again, break the rules of politeness (19) and open new horizons of performative and linguistic interactions which are, perhaps, totally new for the older person. New verbal and non-verbal expressions may develop in order to help increase the pleasure of playing the game. Imagination and simulation of an experience prepare the player to tackle problems in real life situations. Thus, the elements of novelty and creativity flourish. Strategic problem-solving (20), creative thinking, overlapping goal development, involvement with digital literacy practices (21), learning of new skills, and many other cognitive skills become the fruits of such game activities (22–25). Therefore, the aging player may develop skill sets and cognitive capabilities which could be very useful, even essential, in the course of their life. Examples of action- and goal-directed simulations of embodied experience video games include multiplayer games, like World of Warcraft, League of Legends, Minecraft, and Ikariam. The brain activities involved during the course of such games enhance cognition in multiple ways and are thus considered as hormetic. In addition to these, there exist other video games which are more oriented toward literary creativity with prose and poetry, such as the video game Elegy for a Dead World (26).

### Internet and Health

Being cognitively stimulated in a virtual setting is shown to have several health benefits. For instance, playing online action video games affects the plasticity of sensorimotor regions in the gray matter, and improves connectivity between neurons, particularly those involved in attention and experience (27). Use of general video games for 1 h a day improves spatial memory, cognitive control, and complex verbal span (28). These cognitive benefits are not specific to action video games, but other active online experiences may also improve cognition. General use of the Internet has been studied in a variety of settings and initial results show health promoting effects (29) including an increase in life expectancy (30). For instance, a study in Taiwan found that internet use (broadband, wireless, or mobile) is positively associated with quality of life, self-esteem, and reduction in stress (31).

In addition, it was shown that higher levels of Internet use among older people are associated with lower levels of loneliness, improved social support and psychological well-being, and better life satisfaction (32). Web-based interventions which are specific to memory and cognition have a special role to play in cases of mild cognitive impairment and dementia (33).

How does online cognitive stimulation upregulate health? When a mildly stressful information from an online cognitive activity (i.e., information which requires a fast response and instant feedback, such in video games) reaches the neuron, it activates the neuronal stress response which facilitates adaptation to this stressful situation. Neurotransmitters and molecules involved in memory and in cognition are also involved. Such molecules include cyclic guanosine monophosphate, glutamate, leptin, and angiotensin IV. Another marker in hormesis is Heat Shock Proteins (HSP) which is expressed in cases of shock (challenge). Overexpression of HSP is neuroprotective (34).

The risk always exists that excessive use of the Internet, particularly in a way that is repetitive and non-meaningful, may lead to social isolation of the user. In this paper, we highlight the importance of the meaningfulness of information sharing, i.e., information that requires the user to act. In addition, despite concerns that use of online platforms may lead to social isolation, some researchers (35), report that it may, on the contrary, actually decrease loneliness and social isolation. However, older people may experience physical limitations such as arthritis, visual problems, etc. which may impede full use of online activities. The market needs to take into account these age-related limitations.

### Cognitive Challenges May Impact on Physical Health

It is well accepted that physical exercise has cognitive benefits and that cognitive exercise has cognitive benefits [for example, Ref. (36)]. What is less well studied, but nevertheless important, is the *physical* benefits obtained through *cognitive* training. Some research exists in this respect. We know, for example, that while cognitive exercise may increase the speed of information processing (37), it can also improve certain physical health parameters in older people, including vitality, physical functioning, and bodily pain, as well as social and emotional functioning, and instrumental activities of daily living (38). This type of hormetically intense cognitive training not only improves physical functioning but its effects were found to persist for several years (39). A significant number of older people who followed a program of cognitive exercises rated their physical health and quality of life as excellent or very good (40). While clearly more research is needed in this respect, there could be some possible physical health benefits obtained through cognitive stimulation. The hormetic health benefits of cognitive challenges can be augmented by playing online video games or similar engaging activities.

There is growing evidence that these concepts are valid within human groups in the community. D’Orsi et al. (41) have shown that internet use was significantly associated with a lower risk of 10-year mortality, controlling for sex, age, education, cognitive function, functional impairment, and diabetes. In another study, d’Orsi et al. (42), found a specific association between increased use of internet and reduction of dementia risk. Therefore, we could speculate on the potential physical effects obtained through a combination of both physical and cognitive activities, while awaiting further corroboration.

## Conclusion

The increasing presence of online environments, such as action video games and other digital media platforms that share similar characteristics, translate into opportunities for information sharing among the users. In several instances, this information is actually quite meaningful, in the sense that it incites the recipient to act, and it can thus be considered hormetic. We have argued elsewhere (16) that such information may act as a cognitive challenge that upregulates neuronal stress response pathways, the same way as other hormetic challenges and stressors that result in “positive stress” such as physical exercise, heat or cold exposure, radiation, and medication.

Thus, the main points of this paper are several. Cognitive challenges may act along universally encountered hormetic principles, in order to invoke the neuronal stress response. This, as a consequence, upregulates neuronal health and it is likely to result in cognitive health improvement in older people. One example of such cognitive challenge is interaction with online video games, although other challenging cognitive activities may carry similar benefits. We need to encourage increasing use of online action video games for older people.

## Author Contributions

Both contributors have contributed to the conception of the work; MK has discussed biologically related concepts and EK has worked on the acquisition of data and presentation of concepts relating to computer gaming. MK has drafted the work and both have revised it critically; both have agreed the corrections following peer review. Both approve the paper for publication of the content and agree to be accountable for all aspects of the work in ensuring that questions related to the accuracy or integrity of any part of the work are appropriately investigated and resolved.

## Conflict of Interest Statement

The authors declare that the research was conducted in the absence of any commercial or financial relationships that could be construed as a potential conflict of interest.
